# Address Delivered before the Class of the Baltimore College of Dental Surgery, at the Conclusion of the Last Regular Lecture of the Course, for the Session of 1849–50

**Published:** 1850-09

**Authors:** 


*Address delivered before the Class of the Baltimore College of Dental Surgery, at the conclusion of the last regular lecture of the Course, for the Session of* 1849–50. By Chapin A. Harris, M. D., D. D. S., Professor of the Principles and Practice of Dental Surgery.

*Address delivered before the Graduating Class of the Baltimore College of Dental Surgery, at the Tenth Annual Commencement.* By Elisha Townsend, D. D. S.

*Valedictory Address delivered before the Graduating Class of the Baltimore College of Dental Surgery, at the Annual Commencement for the Session of* 1849–50. By S. P. Hullihen, M. D., D. D. S.

*Address to the Society of the Alumni of the Baltimore College of Dental Surgery.* By James Robinson, D. D. S., Dentist to the Royal Free Hospital, &c., London, Eng.

The occasion which called forth the above excellent Addresses, was the Annual Commencement of the Baltimore College of Dental Surgery. This institution, which has been in existence about ten years, is in a highly flourishing condition. So much is it esteemed by the dental profession, not only of the United States, but of other countries, that pupils from abroad are to be numbered among the classes which it annually assembles. How long will it be before the medical schools of our county shall receive a similar tribute to *their* excellence? A quarterly periodical, devoted to dental science and literature, has also been established at Baltimore for several years, published, heretofore, under the auspices of the *American Society of Dental Surgeons*, but, hereafter, as we are informed, to be under the sole direction and editorial management of Prof. Harris. This truly ably conducted Journal, together with the Dental College, have done much to advance the science of dentistry, and to elevate the character of the dental profession. We believe it is conceded that this branch of surgical art has been carried to greater perfection in this, than in any other country; and that, as a profession, dentistry occupies a higher position here—a flattering evidence of the ability and character of a large proportion of American dental practitioners.

The several Addresses whose title pages are given, are alike creditable to the authors, and honorable to the profession of dentistry in this country.

